# Letter of Prof. Butlerow to Prof. Jaeger

**Published:** 1882-04

**Authors:** Ad. Fellger

**Affiliations:** Philadelphia


### Open Letter to Prof. Gustav Jaeger, from Prof. A. Butlerow, Member of the Imperial Academy of Sciences, at St. Petersburg, etc., etc.

[In The Homœopathic Physician, Vol. I, Nos. 1 and 7, we made the following remarks:

"Judging from the proofs which science is rapidly accumulating for us, we may say positively, that in the course of time, allopathy will be forced to admit the correctness of the principles of Homœopathy.

"It will be impossible, hereafter, to find a scientific man, bold enough to deny the efficacy of these highly potentized drugs, without running the risk of being accused of ignorance.

"Since Hahnemann's promulgation of Homœopathy, new discoveries in material science have overthrown many theories and hypotheses of the old school; while on the other hand, these progressive discoveries have in every instance been in harmony with Homœopathy, and have explained clearly the correctness and truth of its principles and teachings."

As these remarks are verified by one of the greatest professors in Russia, a man of European reputation, Prof. Dr. Butlerow, of St. Petersburg, we give below a translation of a letter from him to Prof. Dr. G. Jaeger, of Stuttgart, which we find in January No. (1882), of "Psychische Studien," published in Liepzig.

Ad. Fellger.

### PROF. BUTLEROW'S LETTER.

Highly Honored Colleague:

A few days ago, I had the pleasure of referring, in one of our most widely circulating journals, to the most essential part of the instructive contents of your highly interesting pamphlet, "Neural Analysis." Some remarks preceding this are, in my opinion, calculated to show that the effect of infinitely small doses, and even the increase of this effect corresponding with the dilution, are in not so absolute a contrast to our present knowledge, as is generally taken for granted; according to analogy these effects would rather appear more probable. May I be first permitted to reproduce some of these remarks of mine in a condensed form?

After touching upon the manifestions of hypnotism, metalloscopy, etc., I went on to say: "There are simpler manifestations, which are capable of shaking the high throne upon which matter has been placed. Appertaining to this are, for instance, the well-known observations of Hittorf and Crookes, about the considerable effects of warmth which electricity can call forth when, emanating from the pole, it radiates in a highly attenuated gas. Employing a concave disc as pole, Crookes succeeded in concentrating these electrical rays in such a manner that platinum could be melted. The energy of electricity is in this case conveyed to the platinum through the midst of an almost empty space, and calls forth an extraordinary increase of temperature. What then is here made the vehicle of this energy? This case illustrates clearly how little material substance is required to transmit an excess of power. Nay, even this transmission is only then possible when the quantity of substance has become extremely minimal. We know that when the mass of substance becomes less, the velocity must proportionately become much greater, if the effect is to remain the same. From this point of view, we must in the just mentioned case, assume a velocity exceeding all the limits of our conception, with the most extreme smallness of the quantity of gas, in order to account for the effect. Within our small apparatus we encounter the same infinity which confronts us in the unlimited space of the universe. Here, the infinity of velocity—there, the infinity of space!"

"Hence, we here perceive the same fact, maintained by homœopathists long since: the possibility of producing considerable effects by means of extremely small quantities of substance. The homœopathists even insist upon the effect becoming greater when the amount of substance becomes smaller." * * * "Is that really possible? Asking the analogists, we shall find them inclined to return us an affirmative rather than a negative answer. Indeed, in most cases, we observe that the transition of a substance into an attenuated, finer, simpler condition, is connected with an accumulation of energy in the same; *i.e.*, in just this simpler condition, the substance becomes more effective, more powerful. Thus we notice that the transition from ice to water, from water to aqueous vapor, is accompanied by an absorption of warmth (energy). Steam represents in a certain measure a reservoir of energy, and by separating this energy during the transition of steam into water, a considerable effect, for instance some mechanical labor, can be obtained. We chemists know quite well that in most cases a certain exertion of energy becomes necessary, even in order to decompose a chemically composed substance: the energy, so to speak, disappears in the substance, in order to give it a new, simpler shape, and to remain provisionally latent (potential), in form of chemical energy. Thus, steam can be changed into its components, hydrogen and oxygen, only by a very considerable exertion of energy, and both these elements represent still greater amounts of energy than steam.

"Taking *chemical elements* generally into consideration, we find that in most cases, a more energetic chemical effect belongs to those elements of which we need the least to produce a definite chemical effect (which possess a smaller atomic weight). Since, therefore, in a number of cases, an increase of potential effect is seen to follow attenuation of the condition of substance, why should the possibility of an analogous appearance be denied in cases where the quantities of substance are so small as to be beyond reach of our immediate observation and measurement? Would this not be synonymous with losing sight of the essential circumstance, that the *great* and the *small* are but relative conceptions, as long as infinity in the microcosm as in the macrocosm remains equally inconceivable to us?

"It would even seem to me that, leaving aside these and similar considerations, the simple observations, obtainable by nearly everybody and made already by many, ought to suffice to restrain people from disputing the effect of homœopathic doses. It might almost be asserted that these observations are so little noticed just because they are so ordinary, and of almost daily occurrence. Everybody knows how little of an odorous substance need be present in the space of a room, in order to make a decided, definite impression upon our organs of smell. In the atmosphere of the room, there are in this case everywhere material particles of the smelling substance, and it may frequently happen that no diminution of the quantity of the substance is noticed; this diminution is so small, that we are unable to fix the degree of it by any means at our command. At the same time, it is known how strong the influence of a perceived smell may be when the smelling organism is very sensitive: vomiting, convulsions, fainting, may, for instance, be brought on by a nauseating smell. But, if the influence of infinitely small doses upon the olfactory nerves is subject to no doubt, what cause can we assign to deny the possibility of a similar effect upon our nerves generally? In one case, we become conscious of a perception of smell; in another, although we may remain unconscious of the impression, the latter may exist with as good reason and call forth certain symptoms in the organism. Every one is conscious of the beating of his heart; the peristaltic movements of the bowels, however, are not directly felt, and their existence remains even unknown to many; these movements exist, nevertheless, and perform an important part in the vital process of the organism."

So much of my extracts. I would further ask leave to communicate a few instances, which might not, perhaps, appear uninteresting in view of what you, my highly esteemed colleague, communicate concerning the smell and the taste of homœopathic dilutions. The observations in question were made by one of our well-known homœopathists, Dr. Bojanus,* during his long practice, and published by him. The truthfulness of these observations admits of no doubt:

A woman, healthy, but of delicate constitution, found *Iodine* in higher as well as in lower dilutions not to agree with her. Several times *Iodine* in the potencies 3d to 30th, was experimentally given to her, and each time the woman recognized it by the taste. Yet all this did not appear to Dr. Bojanus to afford sufficiently strong proof, and when the woman happened to be sick of typhus, and was lying senseless in her bed, as a test she was offered *Iodine* in the 30th potency on a lump of sugar. The woman, however, recognized the substance, so disagreeable to her, at once, and spit it out, exclaiming: "Why do you give me such stuff? You ought to know that it does not agree with me!" Upon the woman's recovery she was informed of this case, but could not recollect anything about it.

The second observation of Dr. Bojanus is all the more interesting, since it refers to a plain Russian peasant, who of course could have no idea whatever of medicaments, their doses and virtues. The peasant suffered from a catarrh of the stomach, and was given *Nux vomica*, 12th potency, in the form of powders, each of which contained five to six globules of milk sugar. These powders had to be taken twice a day in the course of the week; seven days after, the patient called to see the physician again, and declared on this occasion that he felt greatly improved. In order not to interfere with the effect of the medicine, and at the same time not to refuse the patient's request, he was given powders again, but this time only such containing pure sugar of milk. Seven days later, the peasant reappeared and declared that the new powders were less effective than the former; "those first ones," he thought, "were bitter, but these other are sweet; give me again of the bitter ones." As a test case, Dr. Bojanus prepared two powders, looking exactly alike; the one contained pure sugar of milk, the other five crushed globules of the 12th potency of *Nux vomica;* when thereupon the pure sugar of

*Dr. C. Bojanus, one of the most eminent homœopathic physicians in Russia, and author of: 1. The History of Homœopathy in Russia; 2. Of Homœopathic Therapeutics; 3. Atlas for Homœopathic Therapeutics; 4. Epilepsy; all of which are very valuable works.

milk was put on the peasant's tongue, he declared at once that this was a sweet powder, at the same time desiring bitter ones. Then he was given the prepared *Nux vomica*, and the peasant at once expressed his joy about having now obtained the desired medicine.

Crookes, according to his own statement, with his extraordinarily attenuated gases and their striking manifestations of power, has nearly reached that remarkable boundary where matter, so to speak, dissolves itself into power. This takes place in a still greater degree in the results of your remarkable observations about the maximum effect of a 2,000th homœopathic potency. Hence the question arises whether we are still justified in attributing a reality to *matter* in itself, if we would abstract the conception of it from that of *power?* Now since we perceive only the effects of power, and only according to these judge about the presence and the qualities of that which we call matter, matter really disappears, and there remains but power or energy. Such a conception of real nature appertains to the philosophers Schopenhauer and Hartmann, and is accepted likewise by many scientists, particularly by A. R. Wallace. We are here confronted with *monism*, as well as with the purely materialistic conception of nature, only energy has assumed the place of matter.

For Schopenhauer and Hartmann, *Will* is the genuine source of power, and Wallace, too, is inclined to share this opinion. Will is, however, for Hartmann, unconscious and void of reason; for Wallace, on the other hand, conscious and endowed with reason. Face to face with the immeasurable amount of misery permeating our terrestrial world, pessimist philosophy, knowing no other world, nor wishing to know such, had needs arrive at the *Unconscious*. But if we ourselves are not limited with our existence to this terrestrial world, the necessity of the unconscious is hereby obviated, and the conscious, reasonable Will assumes its rightful position.

Whether there are forms of existence besides these coarse, material ones, can, for us scientists, be decided in the last resort only by facts. And I now maintain that such *facts* exist; they give forth no uncertain sounds for those willing to listen to them. I would here declare distinctly, that I did not arrive at the acknowledgment of these facts guided, may be, by certain philosophic conceptions, but that, on the contrary, I was compelled to reconcile myself gradually and necessarily with the existence of such facts, and must, accordingly, admit the justification of the above-mentioned conception of nature by Wallace.

Having shown before, that in regard to homœopathic potencies their effect stands in no contradiction to known facts, I will now in a similar way try to answer the question whether the existence of such forms of energy, baffling our direct observation, is probable according to analogy. The answer to this must in my opinion be affirmative. We know that there may exist vibrations of air imperceptible to our organ of hearing, either because they are too slow or too swift for it; we also know that there are waves of light, making no direct impression upon our eye, and become perceptible only by means of some surface fluorescent or sensitive to light. Have we, after this, any right to deny that there are forms of energy which like the aforementioned, are just as real, and yet, under ordinary circumstances, affect none of our feelings? Such forms of energy may remain unknown to us just as well as if they did not exist at all, and this may last until the circumstances appear under which they are perceptible.

Moreover, we know that one form of energy can be transmuted into another (with the maintenance of equivalence according to the law of the preservation of power). This other form may be a just perceptible one for us, whilst the former was not so, and *apparently* matters will look as if a power had originated from nothing. If now, matter be nothing else but a manifestation of power, the same thing may take place with matter; matter would *apparently* arise from nothing and *apparently* be able to be dissolved in nothing, but actually in some form of energy not directly traceable by us. If *Will* is the source of every power, we must from this point of view assume that it can produce what we call matter. If Will is conscious and reasonable, it can do so intentionally. The existence of matter created by the Will might be lasting, but might again be merely temporary.

Now we will ask ourselves whether in this way we have not reached a logical nonsense; is not this the same as maintaining that from nothing something may be made, and *vice versa?* No, by no means! Have we then to abandon the current idea of the eternity of matter? Yes, certainly; but not that of the eternity of energy, or rather of the Will—of the conscious, reasonable Will, the primitive fountain of all power. Shall then, further, the quantity of energy be unchangeable, as this has been asserted till now concerning the quantity of matter (an assumption which under ordinary circumstances is perfectly correct, too), or not? To be sure we cannot conceive that something, therefore energy too, should become nothing; but it is quite as superfluous to speak of the quantity of energy dispersed throughout the universe, as it is superfluous to assume the idea, so familiar to the materialists, of the total quantity of matter in nature. In the one as well as in the other case, the answer is: *Infinity*, therefore, something that entirely excludes the idea of quantity, diminution or increase of it.

We cannot, of course, imagine that, when a labor has been accomplished, a corresponding amount of some form of energy has not been used up for this purpose. But does this likewise hold good even when the labor performed is what we call mental labor? An affirmative answer to this, Dr. Ed. Wegener believes, ought to be given, and likewise Prof. J. Schlesinger. I would not undertake to discuss how far and if at all the assertion appears to be well founded, that the energy used up at some mental labor is not to be found again in any directly perceptible form. As a physiologist, you, my highly honored colleague, will be far more able than I to judge about it; at all events, the circumstance that two scientific thinkers, evidently independent of each other, have arrived at the same result, remains worthy of notice.

If we consider the analogies and conclusions which I have just now mentioned, as established, it is no longer difficult to assume that there exists a supersensual world, which can affect our sensual world. Those manifestations, which my highly esteemed colleague, Prof. Zöllner, considers connected with the existence of his so-called "intelligent beings of four dimensions," no longer offer any fundamental difficulties.

I am, as is known, one of those *many* fortunate, or unfortunate ones (the latter not as to my own personal feelings), who had the opportunity to become firmly convinced of the reality of these so-called "mediumistic" manifestations, as well as of their high importance as to a momentous enlargement of the human view of nature. We who acknowledge these fare precisely as you, highly esteemed colleague, with your discovery: "humbug" and "nonsense"—these are the usual epithets, and that, too, at the hands of those who have not examined at all, or did not wish to examine conscientiously; we have repeatedly noticed that your beautiful rule: "He who condemns without having examined, not only does not deserve the name of an expert, but not even that of an honorable man!" is entirely overlooked. We also "do by no means claim that our assertions should be blindly credited," and believe like you, that "the re-examinations cannot, and may not, be suspended, considering the importance of the matter, lest the men of science should be guilty of a neglect of duty." "Competent specialists try to kill you as well as us by silence. Professor Wundt, of Leipzig, believes himself to be able to ignore your discoveries, and to be obliged to enter the lists against Fichte, Ulrici, Zöllner, etc. All of us have to encounter "*scientific fanaticism.*" Although these two words form a marked contrast, they must nevertheless be often coupled together, because it is not such a rare thing to meet with people who really are less *true* men of science, than priests of a scientific religion invented by themselves, in the dogmas of which, *i.e.* really in the infallibility of their own dear selves, they believe.

In conclusion, allow me, highly esteemed colleague, to express the hope that, in case an opportunity should offer itself to you, to examine mediumistic manifestations, you may not turn from it, and accept the expression of my sincere esteem.*

A. Butlerow.

*St. Petersburg*, 9*th*, 21*st*, 1881.

*Though we cannot indorse all that Prof. Butlerow advances in his letter, we publish it in full at the request of Dr. Fellger.—Editor H. P.

